# Navigating Trifurcated Root Anatomy in Maxillary Premolars: A Detailed Endodontic Case Series

**DOI:** 10.1155/crid/4147927

**Published:** 2026-07-10

**Authors:** Manigandan Kuzhanchinathan, Harsha Nandhini Doraiswamy, Mathan Rajan Rajendran

**Affiliations:** ^1^ Department of Conservative Dentistry and Endodontics, Sri Ramachandra Dental College and Hospital, Sri Ramachandra Institute of Higher Education and Research (SRIHER-DU), Chennai, Tamil Nadu, India

**Keywords:** cone-beam computed tomography, magnification, maxillary premolars, trifurcation, ultrasonics

## Abstract

A foundational grasp of root canal anatomy is vital for achieving predictable treatment and long‐term outcomes in endodontics. Untreated canal is the primary cause of persistent apical periodontitis following endodontic therapy. Maxillary premolars with three roots and canals exhibit a rare but clinically significant anatomical variation characterized by a trifurcation on the root. This feature is frequently overlooked and can contribute to endodontic failure if not properly diagnosed and managed. Recognition of such anatomical variations, combined with the use of radiographic imaging, magnification, and ultrasonics is essential for clinicians to ensure predictable and effective endodontic treatment outcomes. Integration of cone‐beam computed tomography (CBCT) in endodontics significantly enhances the diagnostic precision and accuracy in identifying such complex anatomies. This case series highlights the strategies in endodontic management of maxillary premolars with trifurcation.

## 1. Introduction

The endodontic treatment primarily aims to eliminate and prevent periradicular pathosis [1]. One of the most significant factors in endodontic failure is the presence of undetected or untreated canals, which can serve as reservoir for persistent microbial biofilm [2]. Among the endodontically treated teeth, the second mesiobuccal (MB2) canal of maxillary first molars show the highest prevalence of missed canals, reaching up to 65%. Maxillary premolars also exhibit a notable prevalence of untreated canals, ranging from 4.3% to 13.6% [3]. This range can be due to the ethnic variations, gender predisposition, which could also influence the prevalence of the additional canals and roots [4].

Maxillary premolars typically exhibit two distinct roots or a fused root containing two canals. However, considerable anatomical variability has been reported in this region. According to Vertucci, the maxillary second premolar is unique in its ability to exhibit all eight types of root canal configurations described in their classification system. An anatomical variation occasionally encountered in maxillary premolars is the presence of three separate roots, resulting in a trifurcation of the root structure [5]. This configuration has been documented with an incidence of 5%–6% in maxillary first premolars and approximately 1% in maxillary second premolars [6, 7]. These teeth are often referred to as “mini‐molars,” or “small molars”, due to their morphological resemblance to maxillary molars [8].

Accurate diagnosis of these anatomical complexities is essential for successful endodontic outcomes. Radiographic evaluation plays a pivotal role in detecting the additional canals and aberrant root canal morphology. Radiovisiography (RVG) is one of the digital intraoral imaging techniques that utilizes a conventional dental X‐ray unit along with an electronic sensor and microprocessor to produce images widely used in endodontics [9]. The advantages of RVG over conventional radiography include reduced radiation exposure and immediate image processing [10]. However, as it is a two‐dimensional imaging technique, the diagnostic insights are limited by superimposition of anatomical structures, geometric distortion, and inability to identify aberrant and additional canal system [11].

One of the important radiographic indicators to suspect canal bifurcation or additional root canals is the “fast break” phenomenon, which is characterized by an abrupt disappearance or sudden narrowing of the radiolucent canal space along the root, indicating a division of the main canal into branches or presence of additional canals [12]. In addition, the increased mesiodistal width of the root compared with the crown is also associated with additional root canals [12]. Careful interpretation of fast break, especially when combined with other indicators such as increased mesiodistal root width, helps in early detection of anatomical variations. In such cases, clinical aids such as magnification and ultrasonic tips are used for refining access and locating extra and calcified root canal entrances ensuring precise and controlled removal of dentine [13].

In addition, cone‐beam computed tomography (CBCT) with its high‐resolution, three‐dimensional imaging capabilities has emerged as a superior modality for detecting additional canals, anatomic variations, root resorptions, perforations, and other hard tissue abnormalities [14]. The American Association of Endodontists (AAE) and American Academy of Oral and Maxillofacial Radiology (AAOMR) formulated a collaborative guideline endorsing limited field‐of‐view (FOV) CBCT as the optimal imaging method for assessing intricate root canal morphology and related dental anomalies [15].

While previous case reports have primarily documented the anatomical occurrence of trifurcated maxillary premolars, limited emphasis has been placed on the comprehensive understanding of this anatomical variation, which is essential for improving the treatment outcome. Hence, the aim of this case series is to highlight the significance of preoperative and intraoperative radiographic signs along with selective use of advanced diagnostic modalities like CBCT in successful navigation and endodontic management of maxillary premolars with trifurcation.

This case series was prepared in accordance with the CARE guidelines for case reports [16]. In accordance with the recommendations of the International Committee of Medical Journal Editors (ICMJE), a written informed consent was obtained from all the four patients for the publication of this case series.

## 2. Case Presentation

### 2.1. Case 1

#### 2.1.1. Chief Complaint

A 40‐year‐old male patient with a non‐contributory medical history presented to the outpatient department of Conservative Dentistry and Endodontics, Sri Ramachandra Dental College and Hospital with a complaint of dull throbbing pain in his right upper back region for the past 2 months.

#### 2.1.2. Clinical Examination

Clinical examination revealed Class II dental caries on the mesio proximal surface of Tooth 14 and Class III dental caries on the disto proximal surface of Tooth 13. Tenderness on percussion was evident in relation to Teeth 13 and 14. There was no evidence of mucosal erythema, gingival swelling, or sinus tract in relation to Teeth 13 and 14.

#### 2.1.3. Pulp Sensibility Test

Pulp sensibility testing was performed on both control and test teeth using Endo‐Frost (Coltène/Whaledent GmbH+ Co. KG, Langenau, Germany) sprayed to the cotton pellet (size 2). Application to the mid‐labial surface of control Tooth 23 and 24 elicited immediate response that returned to normal upon removal of the stimulus whereas test teeth (13 and 14) elicited no response.

#### 2.1.4. Radiographic Findings

**Figure 1:**
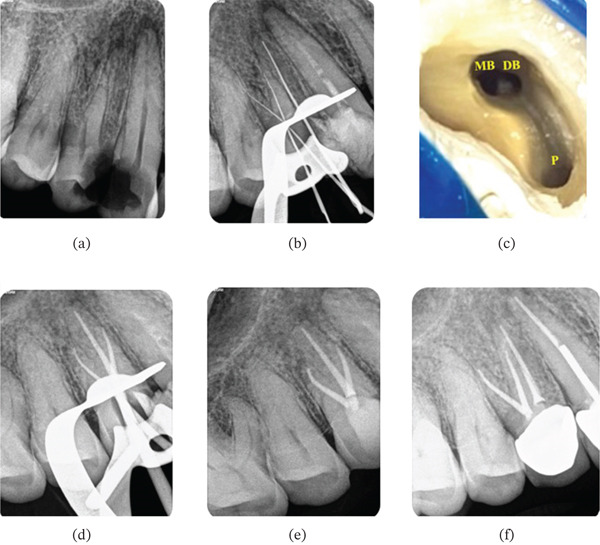
(a) Preoperative radiograph in relation to 14 and 13; (b) working length radiograph in relation to 14; MB = 19 mm, DB = 19 mm, and P = 21 mm; (c) T‐shaped access cavity showing three separate orifices under magnification; (d) master cone radiograph in relation to 14; (e) obturation and core build up radiograph in relation to 14; and (f) 1 year follow‐up radiograph with evidence of periapical healing in 14 and 13 with post endodontic PFM full coverage crowns.

Preoperative radiographic evaluation included an assessment of caries extent, root anatomy, canal morphology, and periapical tissues. RVG with respect to Teeth 13 and 14 revealed radiolucency involving enamel, dentin, and pulp with periapical radiolucency in Tooth 13 and in palatal root of Tooth 14. In addition, Tooth 14 revealed discontinuity of the radiolucency in the mid‐root region (fast break) and mesiodistal dimension of the root exceeds to that of the crown suggesting the presence of additional root/canals (Figure 1a).

#### 2.1.5. Diagnosis

Based on the clinical findings, pulp sensibility testing and radiographic findings; both Teeth 13 and 14 was diagnosed as pulpal necrosis with symptomatic apical periodontitis .

#### 2.1.6. Treatment

Endodontic treatment was performed under dental operating microscope (DOM) (Sanma Lumin Pro, Sanma Medineers Vision Pvt Ltd., Chennai, India). At the initial session, both Teeth 13 and 14 was anesthetized with 2% lignocaine supplemented with 1:100,000 adrenaline (Lignospan, Septodont Healthcare India Pvt. Ltd., Maharashtra, India). After rubber dam isolation and removal of carious lesion, pre‐endodontic build up was done, followed by access cavity preparation. Given the radiographic signs of fast break suggesting an additional canal, access cavity was modified to a T shape [17] in tooth number 14 with ultrasonic tips (ET18D, ETBD—*Satelec Acteon*, France). Three distinct canal orifices were identified on the pulpal floor—one mesiobuccal (MB), one distobuccal (DB), and one palatal (P). Canal patency was confirmed using #10 K‐File (Mani Inc., Utsunomiya, Japan) in all canals.

Working length was determined to be 19 mm in MB and DB, 21 mm in P canals of 14 and 22.5 mm in tooth number 13 with an electronic apex locator (Root ZX, J. Morita, Irvine, CA, United States) and was confirmed radiographically (Figure 1b). Canal preparation was carried out with a crown‐down approach employing rotary nickel titanium (NiTi) instruments driven by an endodontic motor. Root canals were enlarged to size #30.09 (F3, ProTaper Gold, Dentsply Sirona, Ballaigues, Switzerland) for the palatal canal and to size #25.08 (F2, ProTaper Gold, Dentsply Sirona, Ballaigues, Switzerland) for the MB and DB canals of 14. With respect to tooth number 13, canal was enlarged to a size # 40.06 (F4 Protaper Gold, Dentsply Sirona, Ballaigues, Switzerland) Throughout instrumentation, irrigation was performed using 2 mL of 3% sodium hypochlorite (NaOCl, Prime Dental Products, Thane, India). Following the final shaping, 5 mL of 17% ethylenediaminetetraacetic acid (EDTA, Desmear, Anabond Stedman, Chennai, India) irrigation was done for 1 min, after which the canals received a final rinse with 5 mL of 3% NaOCl. The canals were subsequently dried with sterile absorbent paper points and calcium hydroxide intracanal medicament (Ultradent Products Inc., South Jordan, UT, United States) was placed [18]. The access cavity was sealed with interim restorative material (IRM, Dentsply Sirona, Charlotte, NC, United States).

One week later, under the DOM, the teeth were again isolated with a rubber dam, and the interim restoration was removed. Irrigation protocol was carried out similar to the first appointment. The canals were then dried with sterile paper points (Figure 1c) followed by master cone fit radiograph was taken with corresponding F2 and F3 gutta‐percha points in tooth number 14 and F4 gutta‐percha point in tooth number 13 (Dentsply Sirona, Ballaigues, Switzerland) (Figure 1d) and obturation was done in combination with a resin‐based sealer (AH Plus; Dentsply Sirona, Charlotte, NC, United States). The access cavity was subsequently restored using a composite resin material (Figure 1e) (Beautifil II by Shofu Dental Corporation, Osaka, Japan). The patient received full‐coverage porcelain‐fused‐to‐metal (PFM) crowns to rehabilitate the functional integrity and esthetics in both Teeth 13 and 14.

#### 2.1.7. Outcomes

After crown placement, the patient was recalled for routine follow‐up visits over a one‐year period to assess clinical outcomes and periapical healing. Treatment outcome was evaluated based on the Friedman and Mor criteria (2004) combining both the clinical and radiographic signs and symptoms [19]. At each follow‐up period, the patient remained asymptomatic exhibiting no signs of discomfort, tenderness on percussion, or pain on palpation. Radiographic assessment at the one‐year follow‐up further confirmed the signs of periapical healing in Teeth 13 and 14 (Figure 1f).

### 2.2. Case 2

#### 2.2.1. Chief Complaint

A 32‐year‐old female patient with a non‐contributory medical history reported with a complaint of spontaneous pain in her left upper back tooth region for the past 10 days.

#### 2.2.2. Clinical Findings

Clinically, Class II dental caries on the disto proximal surface of tooth number 24 was evident with no tenderness on percussion.

#### 2.2.3. Pulp Sensibility Test

Pulp sensibility testing was performed with Endo‐Frost (Coltène/Whaledent GmbH+ Co. KG, Langenau, Germany) sprayed to the cotton pellet (size 2). An application to the mid‐labial surface of control tooth 14 elicited immediate response that returned to normal upon removal of the stimulus whereas test tooth 24 elicited an immediate response upon application of the stimulus, which persisted more than 15 s after the removal of stimulus.

#### 2.2.4. Radiographic Findings

**Figure 2:**
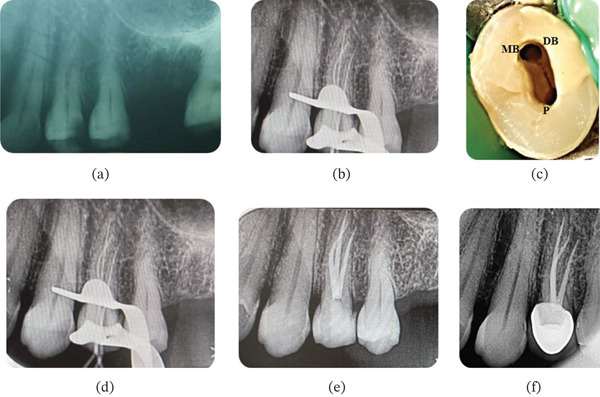
(a) Preoperative radiograph in relation to 24; (b) working length radiograph; MB = 20.5 mm, DB = 19.5 mm, and P = 22 mm; (c) T‐shaped access cavity showing three distinct canal orifices, with the MB and DB orifices located in close proximity under magnification; (d) radiographic confirmation of master cone; (e) obturation and core build up radiograph; and (f) 1 year follow‐up radiograph with post endodontic PFM full coverage crown.

Preoperative radiographic evaluation included assessment of caries extent, root anatomy, canal morphology, and periapical tissues. RVG with respect to Tooth 24 revealed a radiolucency involving enamel, dentin, and pulp with no periapical changes. Additionally, discontinuity of radiolucency (fast break) seen in the mid root region suggestive of the presence of additional root/canals were observed (Figure 2a).

#### 2.2.5. Diagnosis

Based on the clinical findings, pulp sensibility testing and radiographic findings; tooth number 24 was diagnosed as symptomatic irreversible pulpitis with normal periapical tissues.

#### 2.2.6. Treatment

Endodontic treatment was performed under DOM (Sanma Lumin Pro, Sanma Medineers Vision Pvt Ltd., Chennai, India). At the initial appointment, anesthesia was administered using 2% lignocaine combined with 1:100,000 adrenaline (Lignospan, Septodont Healthcare India Pvt. Ltd., Maharashtra, India). After rubber dam isolation and removal of carious lesion, pre‐endodontic build up was done, followed by access cavity preparation. Given the radiographic signs of fast break suggesting an additional canal, access cavity was modified to a T shape [17] with ultrasonic tip (ET18D, ETBD—*Satelec Acteon*, France). Three distinct canal orifices were identified on the pulpal floor –MB, DB, and P canals and patency was confirmed using #10 K‐File (Mani Inc., Utsunomiya, Japan) in all the three canals.

Working length was determined to be 20.5 mm in MB,19.5 mm in DB, and 22 mm in P canals with an electronic apex locator (Root ZX, J. Morita, Irvine, CA, United States) and was confirmed radiographically (Figure 2b). Canal preparation was carried out with a rotary NiTi instruments driven by an endodontic motor. Root canals were prepared to size F3 (#30.09, ProTaper Gold, Dentsply Sirona, Ballaigues, Switzerland) for the P canal and to size F2 (#25.08, ProTaper Gold, Dentsply Sirona, Ballaigues, Switzerland) for the MB and DB canals. Throughout instrumentation, irrigation was performed using 2 mL of 3% NaOCl (Prime Dental Products, Thane, India). Following the final shaping, 5 mL of 17% EDTA (Desmear, Anabond Stedman, Chennai, India) irrigation was done for 1 min, after which the canals received a final rinse with 5 mL of 3% NaOCl. The canals were subsequently dried with sterile absorbent paper points and calcium hydroxide intracanal medicament (Ultradent Products Inc., South Jordan, UT, United States) was placed [18]. The access cavity was sealed with interim restorative material (IRM, Dentsply Sirona, Charlotte, NC, United States).

One week later, under the DOM, the teeth were again isolated with a rubber dam and the interim restoration was removed. Irrigation was carried out similar to the protocol used during the first appointment. The canals were then dried with sterile paper points (Figure 2c) followed by master cone fit radiograph was taken with corresponding F2 and F3 gutta‐percha points (Dentsply Sirona, Ballaigues, Switzerland) (Figure 2d) and obturation was done in combination with a resin‐based sealer (AH Plus; Dentsply Sirona, Charlotte, NC, United States). The access cavity was subsequently restored using a composite resin material (Figure 2e) (Beautifil II by Shofu Dental Corporation, Osaka, Japan). The patient received full‐coverage PFM crown in tooth number 24.

#### 2.2.7. Outcomes

Patient was recalled for routine follow‐up visits over a one‐year period to assess the postoperative healing. Treatment outcome was evaluated based on the Friedman and Mor criteria (2004) combining both the clinical and radiographic signs and symptoms [19]. At each follow‐up period, the patient was completely asymptomatic. Radiographic evaluation at the one‐year follow‐up further confirmed the successful endodontic outcome in tooth number 24 (Figure 2f).

### 2.3. Case3

#### 2.3.1. Chief Complaint

A 37‐year‐old male patient reported with a complaint of fractured restoration along with spontaneous pain in relation to his right upper back tooth region.

#### 2.3.2. Clinical Findings

On clinical examination, fractured restoration (Class II disto proximal) in relation to tooth number 14 was evident with no tenderness on percussion.

#### 2.3.3. Pulp Sensibility Test

Pulp sensibility testing was performed using Endo‐Frost (Coltène/Whaledent GmbH+ Co. KG, Langenau, Germany) sprayed to the cotton pellet (size 2). Application to the mid‐labial surface of control tooth 24 elicited immediate response that returned to normal upon removal of the stimulus whereas the test tooth 14 elicited an immediate response, which persisted for more than 25 s after the stimulus was removed.

#### 2.3.4. Radiographic Findings

**Figure 3:**
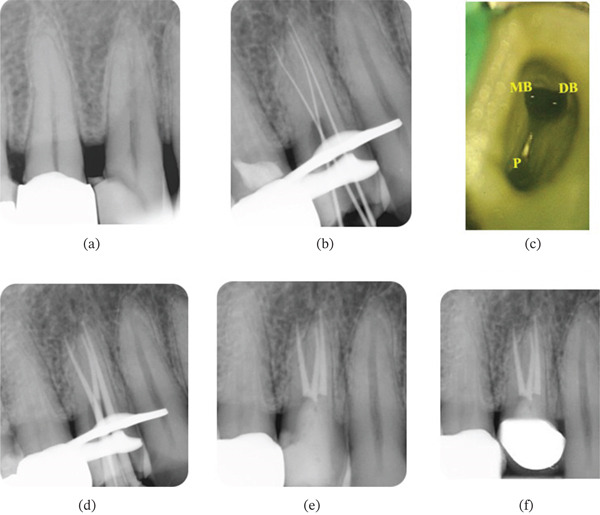
(a) Preoperative radiograph of 14; (b) working length radiograph; MB = 19 mm, DB = 18.5 mm, and P = 20.5 mm; (c) access cavity with a T‐shaped configuration showing three orifices under magnification; (d) master cone radiograph; (e) post obturation and core build up radiograph; and (f) follow‐up radiograph at the end of 1 year with PFM full coverage crown.

Preoperative radiographic evaluation included assessment of caries extent, root anatomy, canal morphology, and periapical tissues. RVG examination of tooth number 14 revealed radiolucency beneath the radiopacity approximating the pulp on the disto proximal side. Additionally, a discontinuity of radiolucent line was evident in the mid‐root region (fast break) along with a wider mesiodistal dimension of the root compared with that of the crown portion indicated the possible presence of additional root/canal (Figure 3a).

#### 2.3.5. Diagnosis

Based on the clinical findings, pulp sensibility testing and radiographic findings; tooth number 14 was diagnosed with symptomatic irreversible pulpitis with normal periapical tissues.

#### 2.3.6. Treatment

Endodontic treatment was carried out under a DOM (Sanma Lumin Pro, Sanma Medineers Vision Pvt Ltd., Chennai, India). At the initial visit, the tooth was anesthetized with 2% lignocaine supplemented with 1:100,000 adrenaline (Lignospan, Septodont Healthcare India Pvt. Ltd., Maharashtra, India). Following rubber dam isolation, the fractured restoration and secondary caries were removed. Pre‐endodontic build‐up was done, followed by access cavity preparation. Based on the radiographic evidence of a fast break suggestive of an additional canal, the access cavity was modified to a T‐shaped [17] design using ultrasonic tips (ET18D, ETBD—Satelec Acteon, France).

MB, DB, and P canal orifices were identified on the pulpal floor and canal patency was confirmed in all canals using#10 K‐file (Mani Inc., Utsunomiya, Japan). Working length was determined using an electronic apex locator (Root ZX, J. Morita, Irvine, CA, United States) and confirmed radiographically (Figure 3b). The working lengths were 19 mm in the MB canal, 18.5 mm in the DB canal, and 20.5 mm in the P canal.

Root canal shaping was performed using NiTi instruments driven by a rotary endodontic motor. Root canals were prepared to size F3 (#30.09, ProTaper Gold, Dentsply Sirona, Ballaigues, Switzerland) for the P canal and to size F2 (#25.08, ProTaper Gold, Dentsply Sirona, Ballaigues, Switzerland) for the MB and DB canals. Between each instrumentation cycle, irrigation was carried out with 2 mL of 3% NaOCl (Prime Dental Products, Thane, India). After completion of shaping, the canals were irrigated with 5 mL of 17% EDTA (Desmear, Anabond Stedman, Chennai, India) for 1 min, followed by a final rinse with 5 mL of 3% NaOCl.

The canals were then dried with sterile absorbent paper points and calcium hydroxide intracanal medicament (Ultradent Products Inc., South Jordan, UT, United States) was placed [18]. Finally, the access cavity was sealed with an interim restorative material (IRM, Dentsply Sirona, Charlotte, NC, United States).

One week later, the tooth was re‐isolated under the DOM with a rubber dam and the interim restoration was removed. Irrigation was performed following the same protocol as the first visit. The canals were dried with sterile paper points (Figure 3c), and a master cone radiograph was taken using corresponding F2 and F3 gutta‐percha points (Dentsply Sirona, Ballaigues, Switzerland) (Figure 3d). Obturation was completed with corresponding gutta‐percha points and resin‐based sealer (AH Plus; Dentsply Sirona, Charlotte, NC, United States). The access cavity was restored with composite resin (Beautifil II by Shofu Dental Corporation, Osaka, Japan) (Figure 3e). Post‐endodontic rehabilitation included placement of a full‐coverage PFM crown.

#### 2.3.7. Outcomes

After crown placement, the patient was recalled for follow‐up over 1 year to evaluate clinical outcomes and periapical healing. Treatment outcome was evaluated based on the Friedman and Mor criteria (2004) combining both the clinical and radiographic signs and symptoms [19]. At each follow‐up period, patient remained asymptomatic, with no discomfort, tenderness on percussion, or swelling. Radiographic evaluation at 1 year confirmed successful endodontic management (Figure 3f).

### 2.4. Case 4

#### 2.4.1. Chief Complaint

A 35‐year‐old female patient reported with spontaneous pain in relation to her right upper back tooth region.

#### 2.4.2. Clinical Findings

On clinical assessment, Class II caries was present on the disto proximal surface of tooth number 15 along with tenderness on percussion was evident.

#### 2.4.3. Pulpal Sensibility Test

Pulp sensibility testing using Endo‐Frost (Coltène/Whaledent GmbH+ Co. KG, Langenau, Germany) sprayed to the cotton pellet (size 2) placed on mid‐labial surface of control Tooth 25 elicited immediate response that returned to normal upon removal of the stimulus whereas the test Tooth 15 resulted in lingering response that lasted more than 25 s upon removal of the stimulus.

#### 2.4.4. Radiographic Findings

**Figure 4:**
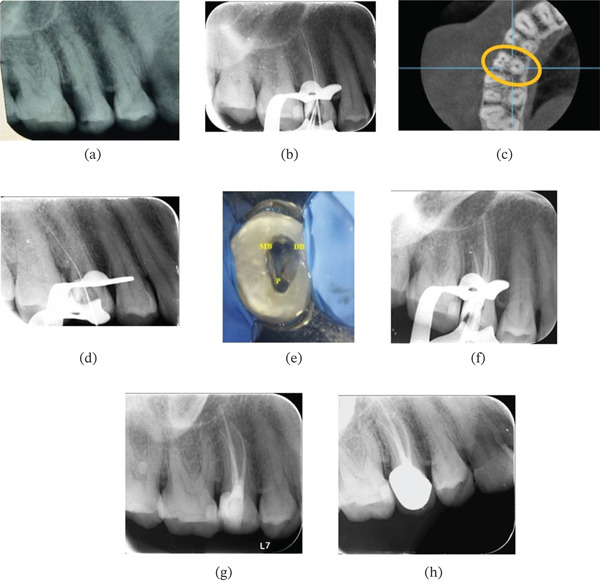
(a) Preoperative radiograph of 15; (b) working length radiograph—MB = 19 mm and P = 21 mm; (c) axial view of CBCT showing the presence of three distinct canals; (d) working length radiograph; DB = 19.5 mm; (e) T‐shaped access cavity showing three orifices under magnification; (f) master cone radiograph; (g) obturation and core build up radiograph; and (h) 1 year post treatment follow‐up radiograph with PFM full coverage crown.

Preoperative radiographic evaluation included assessment of caries extent, root anatomy, canal morphology, and periapical tissues. RVG analysis of tooth number 15 revealed a radiolucent lesion involving the enamel, dentin, and pulp on the disto proximal surface. Additionally, a discontinuity in the radiolucent outline in the mid‐root region suggestive of a fast break, along with a mesiodistal root dimension greater than that of the crown, indicated the possible presence of additional root/canal (Figure 4a).

#### 2.4.5. Diagnosis

Based on the clinical findings, pulp sensibility testing and radiographic findings; tooth number 15 was diagnosed with symptomatic irreversible pulpitis with normal periapical tissues.

#### 2.4.6. Treatment

Endodontic treatment was performed under a DOM (Sanma Lumin Pro, Sanma Medineers Vision Pvt Ltd., Chennai, India). After administering local anesthesia using 2% lignocaine with 1:100,000 adrenaline (Lignospan, Septodont Healthcare India Pvt. Ltd., Maharashtra, India), rubber dam isolation was achieved. The carious lesion was removed, and a pre‐endodontic build‐up was done, followed by access cavity preparation. Initially, only the MB and P canals were located and the working length was determined as 19 mm in MB and 21 mm in P canals using an apex locator (Root ZX, J. Morita, Irvine, CA, United States) and was subsequently confirmed radiographically (Figure 4b). However, because of persistent suspicion of an additional canal, CBCT scanning was done and confirmed the presence of a DB canal in the axial section (Figure 4c).

At the subsequent appointment, after administering local anesthesia and achieving rubber dam isolation, troughing of dentin was done slightly below the pulpal floor on the distal aspect using ultrasonic tips (ET18D, ETBD—Satelec Acteon, France) revealed the DB canal orifice. The working length of the DB canal was then determined as 19.5 mm using the apex locator and verified radiographically (Figure 4d). Canal preparation was subsequently performed to size F3 (#30/.09, ProTaper Gold, Dentsply Sirona, Ballaigues, Switzerland) in the P canal and to size F2 (#25/.08, ProTaper Gold, Dentsply Sirona, Ballaigues, Switzerland) in MB and DB canals. During instrumentation, irrigation was performed using 2 mL of 3% NaOCl (Prime Dental Products, Thane, India). After completion of shaping, final irrigation was carried out using 5 mL of 17% EDTA (Desmear, Anabond Stedman, Chennai, India) for 1 min, followed by a final rinse with 5 mL of 3% NaOCl. The canals were then dried using sterile absorbent paper points, and calcium hydroxide intracanal medicament (Ultradent Products Inc., South Jordan, UT, United States) was placed [18]. The access cavity was sealed with an interim restorative material (IRM, Dentsply Sirona, Charlotte, NC, United States).

One week later, the tooth was again isolated under the DOM using a rubber dam, and the interim restoration was removed. Irrigation was performed following the same protocol as in the previous visit. The canals were dried with sterile paper points (Figure 4e), and a master cone fit radiograph was taken using corresponding F2 and F3 gutta‐percha points (Dentsply Sirona, Ballaigues, Switzerland) (Figure 4f). Obturation was then completed using the corresponding gutta‐percha points in combination with a resin‐based sealer (AH Plus; Dentsply Sirona, Charlotte, NC, United States). Finally, the access cavity was restored with composite resin (Beautifil II by Shofu Dental Corporation, Osaka, Japan) (Figure 4g). The patient subsequently received a full coverage PFM crown in tooth number 15.

#### 2.4.7. Outcomes

Treatment outcome was evaluated based on the Friedman and Mor criteria (2004) combining both the clinical and radiographic signs and symptoms [19]. At each follow‐up period, the patient was clinically asymptomatic with no discomfort, percussion tenderness, or swelling. Radiographic evaluation confirmed satisfactory periapical healing and successful endodontic treatment outcome in tooth number 15 (Figure 4h).

## 3. Discussion

The present case series highlights the importance of radiographic interpretation and clinical aids in identifying extra roots/canals in maxillary premolars. The key findings include the consistent presence of radiographic indicators such as “fast break” in all the cases and increased mesiodistal root width, which created the suspicion of additional canals. The integration of ultrasonics with magnification, and selective use of CBCT facilitated successful identification and management of maxillary premolars with trifurcated anatomy.

Persistent AP in root canal treated teeth is predominantly attributed to undetected canals, inadequate instrumentation, incomplete debridement, and deficiencies in coronal or apical sealing [20]. Previous clinical studies showed that 83%–98% of persistent AP cases in root canal treated teeth were due to untreated canals [2, 21, 22]. Corroborating this, it was found that teeth with undetected canals are 4.38–6.25 times more prone to develop periapical pathology [2, 21]. The occurrence of missed canals is well documented in teeth requiring endodontic retreatment, with the maxillary arch exhibiting a higher incidence than the mandibular arch [3]. Hoen and Pink reported that 42% of nonsurgically retreated teeth harbored missed canals, which, if initially sterile, can later serve as a nidus for reinfection [23]. These findings, substantiated by multiple studies underscore the paramount importance of comprehensive canal detection, meticulous debridement, and optimal sealing in achieving long‐term endodontic success [2, 21, 22].

The occurrence of a maxillary first premolar with trifurcation is relatively uncommon, with a reported incidence of 5%–6% while in maxillary second premolars, it is even lower at 0.4%–1% [4–7]. Ethnic background significantly influences root canal anatomy and it was found that the trifurcated maxillary premolar is less common in Asian populations than in European or African groups, with its prevalence in Asians documented at only 0.3%–0.5% [4]. In addition, sexual dimorphism plays a significant role in root and canal morphology, with males exhibiting a greater predisposition toward trifurcated maxillary premolars compared with females. This phenomenon is attributed to increased enamel and dentin deposition regulated by the Y chromosome, resulting in larger teeth and more complex root canal configurations [4].

In the present case series, all the four patients were from Indian population. Though rare in Asian population, clinicians must have a thorough knowledge about the anatomical variations associated with maxillary premolars. Among the four patients, two patients were male and two were female, which reflects this anatomical variability.

According to Sieraski et al., a maxillary premolar is more likely to present with three roots when the mesiodistal dimension of the mid‐root is equal to or exceeds the width of clinical crown [8]. Martínez et. al investigated the effect of varying X‐ray tube angulation on detecting premolar root canal systems and demonstrated that altering the horizontal angulation of 20°–40° enhanced the detection of additional canal/aberrant anatomy canal anatomy, showing better correlation with the true internal anatomy [24]. In the present case series, RVG is used as a radiographic modality for initial assessment. Compared with conventional film‐based radiography, RVG offers additional features including reduced radiation exposure (up to 70%–80%), rapid image acquisition, contrast enhancement, magnification, and negative‐to‐positive inversion [10]. RVG offers image enhancement using up to 256 shades of gray [25].

Based on the present four cases, a stepwise diagnostic approach is proposed as follows: (1) preoperative radiograph and careful evaluation of the radiographic indicators like the fast break and the increased mesiodistal root width; (2) when these indicators are present, modification of the access cavity to a T‐shaped design, which is done by troughing with ultrasonics and exploration of thee pulpal floor under magnification; (3) when the extra canal remains undetected even after the application of all these aids, selective limited FOV CBCT is advised to confirm the presence and location of the additional canal as it aligns with the AAE/AAOMR guidelines. CBCT serves as a key tool for visualizing detailed root canal anatomy, offering the ability to examine numerous planar views of a single tooth [26, 27]. The sensitivity and specificity of CBCT in locating the additional root/canal with CBCT is about 94% and 93.1%, respectively [28]. A definitive difference must be drawn in selecting between the diagnostic roles of RVG and CBCT. In Cases 1, 2, and 3 the radiographic indicators were clearly evident, this is combined with modified T‐shaped access, magnification, and ultrasonic troughing and this approach was sufficient to locate all three canal orifices without any three‐dimensional advanced radiographic imaging. This confirms that RVG could be considered as the first line of diagnostic modality when clinical aids are used in combination.

However, in Case 4, in spite that same indicators were present but still, DB canal orifice could not be traced clinically. This reflects the limitation of two dimensional RVG.RVG could suggest the presence of additional canal, the precise buccolingual location, depth, or spatial relationship of the orifice relative to adjacent canals could not be detected with RVG, which was important in Case 4. In such scenario, CBCT resolved this by revealing the DB canal’s orientation and proximity to the MB canal in the axial section, which enabled the targeted clinical access at the subsequent visit and successful location of the DB canal. This is supported by previous studies, which proved that CBCT had a better prevalence of locating the extracanal when using as a diagnostic aid [29]. Therefore, CBCT should not be used routinely, but reserved as a definitive diagnostic tool only after conventional radiographic and clinical methods have been systematically exhausted, consistent with the ALARA principle and current professional guidelines [15].

The typical presentation of the canals in trifurcated maxillary premolars includes MB, DB, and P canals. A deviation from the expected buccopalatal alignment of the pulp chamber further supported the suspicion of an additional canal. With the help of all these findings, the access cavity design was modified to a T‐shaped design to aid in better exploration of the root canal orifices [17].

Magnification and ultrasonic tips further improved the clinical outcomes in endodontics by enabling precise access refinement and enhancing the identification of additional canals [30, 31]. They are particularly effective in locating calcified canals and facilitating the removal of pulp stones, thereby optimizing canal debridement and overall treatment efficacy. In the present case series, ultrasonic tips were utilized for controlled troughing along the pulpal floor and selective dentin removal, which improved access to the root canals while preserving the remaining tooth structure.

All the patients were treated under rubber dam isolation and magnification to ensure the endodontic treatment standards. The MB and DB canals were instrumented up to size #25, while the P canal was prepared to size #30, ensuring effective cleaning, shaping, and irrigation [32]. Given the potentially increased risk of flare‐ups, multiple‐visit root canal treatment was preferred in all the cases due to the anatomical complexities [33]. During the interappointment period, calcium hydroxide was placed as an intracanal medicament to aid in the effective reduction of the microbial load and to improve periapical healing outcome [18]. Obturation was completed using resin sealer in conjunction with the corresponding gutta‐percha points, followed by the placement of a composite restoration and full coverage PFM crown to establish an optimal coronal seal and prevent the microleakage.

The limitations of this present case series were that it included only four cases and had a follow‐up period of 1 year. Even though CBCT was taken for only one of the included cases, the overall clinical outcomes were satisfactory. Unlike previous reports that primarily described the anatomical occurrence of trifurcated premolars, this case series emphasizes on integrating the radiographic indicators along with clinical strategies in framing an enhanced treatment approach for management of maxillary premolars with trifurcated anatomy.

While encountering endodontic treatment of maxillary premolars, it is quite critical to evaluate and note the radiographic indicators, which can denote trifurcation. When these are found, the access should be sufficiently modified to locate all the canal orifices along with aids like magnification and ultrasonics. When all these methods failed to help in locating the canal orifices, limited FOV CBCT can be selectively employed.

## 4. Conclusion

A comprehensive understanding of root canal anatomy is essential for the success of endodontic treatment, especially in teeth with anatomical complexities. This case series reinforces a structured treatment approach including the radiographic indicators, access design, magnification along with ultrasonics and advanced imaging technique in successful management of maxillary premolars with trifurcated anatomy.

## Funding

No funding was received for this manuscript.

## Conflicts of Interest

The authors declare no conflicts of interest.

## Data Availability

The data that support the findings of this study are available from the corresponding author upon reasonable request.
